# Shaping the biology of citrus: II. Genomic determinants of domestication

**DOI:** 10.1002/tpg2.20133

**Published:** 2021-08-31

**Authors:** Daniel Gonzalez‐Ibeas, Victoria Ibanez, Estela Perez‐Roman, Carles Borredá, Javier Terol, Manuel Talon

**Affiliations:** ^1^ Instituto Valenciano de Investigaciones Agrarias (IVIA) Carretera Moncada CV‐315, Km 10 Valencia 46113 Spain

## Abstract

We performed genomic analyses on species and varieties of the genus *Citrus* to identify several determinants of domestication, based on the pattern of pummelo [*Citrus maxima* (Burr. f) Merr] and mandarin (*Citrus reticulata* Blanco) admixture into the ancestral genome, as well as population genetic tests at smaller scales. Domestication impacted gene families regulating pivotal components of citrus flavor (such as acidity) because in edible mandarin varieties, chromosome areas with negative Tajimas values were enriched with genes associated with the regulation of citric acid. Detection of sweeps in edible mandarins that diverged from wild relatives indicated that domestication reduced chemical defenses involving cyanogenesis and alkaloid synthesis, thus increasing palatability. Also, a cluster of *SAUR* genes in domesticated mandarins derived from the pummelo genome appears to contain candidate genes controlling fruit size. Similarly, conserved stretches of pure mandarin areas were likely important as well for domestication, as, for example, a fragment in chromosome 1 that is involved in the apomictic reproduction of most edible mandarins. Interestingly, our results also support the hypothesis that various genes subject to selective pressure during evolution or derived from whole genome duplication events later became potential targets of domestication.

## Core Ideas

- We identified genomic determinants of citrus biology targeted by domestication.
- Apomixis and fruit size appear to be key in citrus domestication.
- Domestication affected gene families contributing to citrus flavor.
- Combination of evolution and domestication studies helps to elucidate such genomic determinants.

**Abbreviations**
FST: fixation index
PBS: Population Branch Statistic
PCA: principal component analysis
SAF: site allele frequency
SFS: site frequency spectrum
SNP: single nucleotide polymorphism
TCA: tricarboxylic acid cycle
WGD: whole genome duplication

## 1 INTRODUCTION

Domestication has produced major genetic differences between wild species and cultivated plants through breeding (Doebley et al., 2006). Studies of evolution and subsequent domestication can shed light on the lability of genomes and the adaptation of genes to environments. These investigations may also help to identify specific gene families or members within each family that greatly influence the biology of the species and therefore constitute valuable resources for plant breeding programs.

Studies of plant domestication typically involve analyzing allele frequencies in search of genome footprints of human artificial selection, including (a) divergence from wild relatives and (b) reduction of genetic diversity (genome sweeps) among a population of domesticated varieties (Hohenlohe et al., 2010). The Population Branch Statistic (PBS), which is based on the popular pairwise fixation index (Fst) distances, can be effectively used for identifying genes with the highest dissimilarity between a certain population and two others, as an improvement over simple Fst analysis. Such approach has been successfully applied elsewhere—for example, to analyze adaptation to high altitude in Tibetans relative to Chinese and European humans (Yi et al., 2010) and to analyze domestication in maize (*Zea Mays* L.), comparing teosinte with two populations of cultivars (da Fonseca et al., 2015). On the other hand, alterations of genetic diversity on genome areas that depart from a neutral model of evolution due to selective pressure can be measured by Tajima's D statistic (Nielsen, 2001; Tajima, 1989). In this case, positive values may denote both balancing selection and the effect of introgression, whereas negative values are typically inferred as an indication of selection by domestication.

Citrus domestication has been a focus of several reviews in recent years (Deng et al., 2020; Rao et al., 2021). Research is mainly focused on the agronomic traits of the fruit, such as seedlessness, yield, flavor, juiciness, firmness, acidity, peel color, fruit growth, and ripening. Asexual reproduction through nucellar embryony of the seed (i.e., apomixis) is another highly appreciate trait as it produces offspring genetically identical to the mother parental preventing the segregation of desirable characters. Polyembriony has recently been associated with the *CitRWP* gene (Wang et al., 2017), whereas repression of this gene abolishes nucellar embryony. Several candidate genes have been proposed to control other attractive domestication traits, such as *FT* (early flowering), *CitPH1* and *CitPh5* (acidity), Ruby and Noemi (anthocyanin accumulation), CCD4 (carotenoid accumulation), among others (revised in Rao et al., 2021). It was also reported that pathogen and insect tolerance and resistance of domesticated citrus has generally declined compared with wild relatives, as is clearly exemplified in recent decades with the devastating bacterial diseases known as Huanglongbing (caused by *Candidatus* Liberibacter africanus, *C*. Liberibacter americanus, *C*. Liberibacter asiaticus) and citrus canker [caused by *Xanthomonas citri* subsp. *citri* (ex Hasse) Gabriel et al.] (Rao et al., 2021). Thus, citrus domestication appears to be mostly characterized by a reduction in fruit acidity, anthocyanin accumulation, and disease tolerance, while the apomictic behavior was incorporated as a general rule to all edible varieties.

Domesticated citrus are genetic admixtures derived from three ancestral pure species: pummelo [*Citrus maxima* (Burr. f) Merr], mandarin (*Citrus reticulata* Blanco), and citron (*Citrus medica* L.). As observed in the hundreds of current varieties of mandarins, oranges [*Citrus x sinensis* (L.) Osbeck], grapefruits (*C. x paradisi* Macfadyen), and lemons [*C. x limon* (L.) Burm. F.], the pummelo introgression in the mandarin genome is a general feature of all edible cultivars (Wu et al., 2014;2018), an observation indicative that the integrated pummelo fragments included traits that were fundamental to domestication and consequently selected for and fixed during the initial episode of citrus domestication. Other improvements were likely fixed by new crosses between the ancestral species and/or derived hybrids or admixtures (Talon et al., 2020). Thereby, mandarins can be classified according to their pummelo content as a proxy of domestication degree: mandarins of Type I are pure mandarins that do not carry pummelo introgression, mandarins of Type II show low introgression levels (0.3–7.0%), whereas mandarins of Type III contain higher percentages (12–23%) (Wu et al., 2018). In this work, we applied the PBS and Tajima's D statistics to domesticated and wild populations of mandarin genomes based on these pummelo contents and/or their palatability to identify domestication signatures. Because domestication is often considered an evolutionary process, distinguishing between forces driving natural evolution and those governing artificial selection is rather challenging from methodological and conceptual stand points. Thus, we have also investigated the connection between genomic determinants identified in ancestral species during the evolution of the genus (Gonzalez‐Ibeas et al., 2021) and their role on domestication, which revealed that some evolutionary determinants are eventually related to agricultural traits.

**Core Ideas**

- We identified genomic determinants of citrus biology targeted by domestication.
- Apomixis and fruit size appear to be key in citrus domestication.
- Domestication affected gene families contributing to citrus flavor.
- Combination of evolution and domestication studies helps to elucidate such genomic determinants.

## 2 MATERIALS AND METHODS

### 2.1 Single nucleotide polymorphism identification

DNA HiSeq Illumina reads from whole genome sequencing projects were retrieved from repositories (Supplemental Table S5) and quality filtered with custom Python scripts (Phred score >30, read length 100 bp). Trimmed reads were mapped on the clementine haploid reference genome (v1.0, Phytozome; Goodstein et al., 2012) with bwa‐mem (v0.7.15‐r1140) (Li & Durbin, 2009), setting ‐r 1.2 option. Only reads of mapping quality >10 and uniquely mapped on the genome were used from BAM files. Polymerase chain reaction duplicates were removed using Picard MarkDuplicates (v1.139) (http://broadinstitute.github.io/picard/) deployed in the Genome Analysis Toolkit (GATK; Van der Auwera et al., 2013). Single nucleotide polymorphisms (SNPs) were called with GATK HaplotypeCaller (v3.8‐1‐0) (Van der Auwera et al., 2013) with default parameters and with subsequent filtering as set in (Wu et al., 2018). Only bi‐allelic SNPs are used in this analysis. Variant Call Format (VCF) files were processed with the GATK tools for additional filtering, including read base quality score (Q ≥ 30), quality by depth (QD ≥ 2.0), Phred‐scaled *P*‐value using Fisher's exact test to detect strand bias (FS < 60), read mapping quality (MQ > = 40), Z‐score From Wilcoxon rank sum test of Alt vs. Ref read mapping qualities (MQRankSum > −12.5), Z‐score from Wilcoxon rank sum test of Alt vs. Ref read position bias (ReadPosRankSum > −8.0). VCFtools (v0.1.13) (Danecek et al., 2011) and custom Python scripts were also used to filter VCFs by position.

### 2.2 Tajima's D statistic estimation

Tajima's D statistic was calculated with ANGSD (v0.928) (Korneliussen et al., 2014) with htslib (v1.9; http://www.htslib.org/). BAM files previously generated of aligned DNA reads on the clementine reference genome were used as input. Reads with mapping quality <30 and Phred score <30 were filtered out. Site allele frequency (SAF) likelihoods based on individual genotype likelihoods assuming the Hardy‐Weinberg equilibrium were calculated first, and the site frequency spectrum (SFS) was calculated with realSFS. The folded SFS by setting ‐fold 1 for realSFS with the clementine genome as reference was used. Site frequency spectrum output was used as prior SFS with the ‐pest argument of ANGSD to calculate the posterior site allele frequencies and the thetas (population scaled mutation rate) and the final Tajima's D. Clementine genome was analyzed in windows of 50‐Kb per 10‐Kb steps with thetaStat program of ANGSD.

### 2.3 PBS estimation

Population Branch Statistic was calculated with ANGSD (v0.928) (Korneliussen et al., 2014) with htslib (v1.9; http://www.htslib.org/). The Fst was first calculated with realSFS fst index from ANGSD as a measure of population differentiation from polymorphism data. BAM files were used as input in three independent runs of samples categorized according to the amount of introgressed pummelo content (Type I, II, and III mandarins). Reads with mapping quality <30 and Phred score <30 were filtered out. Similarly to Tajima's D calculation, SAF likelihoods were calculated first, and the SFS was calculated with ANGSD's RealSFS for each of the three mandarin populations. The three files were used as input of the RealSFS fst program to output PBS for each population, and “stats2” option was used to calculate the index per genome windows of 50 Kb in 10 Kb steps.

### 2.4 Analysis of GO categories based on per gene Tajima's D and PBS indexes ranking

Values of both Tajima and PBS indexes were calculated per gene taking into account only the coding regions (CDS, neither intron nor UTR sequences were used) with independent ANGSD runs for each gene. Genes were ranked according to their values, and overrepresented GO categories in gene sets in relation to all GO terms from the whole set of annotated genes (GO annotation background) were identified with the logistic model of the Babelomics suit (v5.2.5) (Alonso et al., 2015). This model is of special application for this analysis since Tajima and PBS indexes do not provide a clear predefined value to be set as cut‐off for significance. For example, Tajima's values of +1 and −1 are used as threshold elsewhere, but no generalized consensus exist. The logistic model takes as input a list of genes ranked by their values of a continuous variable and allows the partitioning of the list to detect gene sets that are consistently associated with high or low values of that variable (Alonso et al., 2015). Thus, this model avoids the usage of subjective predefined thresholds for the domestication indexes used.

### 2.5 Identification of admixture among mandarins

Admixture was estimated with NGSadmix in pairwise comparisons of the mandarins listed in Supplemental Table S5. Mangshan mandarin was taken as reference of pure mandarin. Each sample combination was analyzed in independent runs. Admixture proportion was calculated in windows of 100 Kb and 50 Kb of step overlap. Genotype likelihoods in .beagle format were generated with ANGSD by using BAM files as input and were used as input for NGSadmix, setting the minimum minor allele frequency to 0.05 and number of clusters to ‐K 2 per sample combination. For each sample combination we calculate admixture proportion of two mandarins; thereby the theoretical expected areas are 100% (1.0) mandarin A, 100% (1.0) mandarin B, or 50%:50% (0.5) mandarin A/mandarin B. Due to chimerism (highly abundant in citrus, as we reported [Terol et al., 2015]), distribution of somatic mutations in sectors, as usual in perennials (Burian et al., 2016), failure in SNP identification, wrong window size or any other source of error, a few areas do not fit these expected proportions. Therefore, only genome windows where admixture proportion ranged from 0.4 to 0.6 were considered and accounted. Heterozygosity was calculated as the percentage of heterozygous SNPs relative to total identified SNPs in windows of 2 Kb along the clementine reference genome. Results were plotted with Circos (v0.69‐8) (Krzywinski et al., 2009).

### 2.6 Other analysis

For principal component analysis (PCA), BAM files were used as input for ANGSD to generate the covariance matrix. The matrix was processed with the eigen() R function and plotted with R. ANGSD was also used to generate an IBS matrix for doing a multidimensional scaling (MDS) plot with the cmdscale() R function. For identification of SNPs associated to components, SNPs were called by ANGSD with the ‐doGeno option and the genotype matrix was used as input for PCAdapt (v4.1.0) (Luu et al., 2017). *P*‐values were corrected with the qvalue R package (α = 0.1) during the screening.

*Citrus sinensis* genome areas previously identified involved in mandarin domestication (Wang et al., 2018) were mapped on the clementine reference genome (v1.0, Phytozome) with BLAST (v2.6.0+) (Altschul et al., 1990). Hits longer than 1 Kb, E‐value <1e‐05 and sequence identity > 90% were retrieved and mapped on the clementine genome.

Overlap of pummelo introgressed genome areas potentially involved in fruit size were identified and stacked by BLAST comparison of previously reported GFF3 genome coordinates from independent accessions (Wu et al., 2018). Phylogenetic analysis of protein sequences was performed with MUSCLE (v3.8.31) (Edgar, 2004) for protein sequence alignment, RaxML (v8.2.11+dfsg‐1) (Stamatakis, 2014) for tree generation and FigTree (v1.4.4) (http://tree.bio.ed.ac.uk/software/figtree/) for tree visualization.

### 2.7 RESULTS

### 2.8 Selective genome sweeps in domesticated varieties and quantification of their divergence from wild relatives

We compared SNP data in a set of 19 mandarins to study the genetic diversity at population level in order to detect both genetic divergence and selective sweeps. Population Branch Statistic was calculated on the three types of mandarins (I, II, and III; Wu et al., 2018) to find genes showing the greatest differences in edible Type III modern accessions relative to other relatives. For Tajima's index calculation, a set of domesticated accessions was used (Supplemental Table S5). The distribution of indexes across the entire genome revealed a complex and uneven pattern. Overall, Tajima and PBS values correlated with the introgression rate (Supplemental Figure S1a2), as expected. Chromosomes 4 and 5 revealed the most negative Tajima's and lowest PBS indexes, suggesting that mandarin areas of these chromosomes contributed more to domestication than pummelo.

**FIGURE 1:**
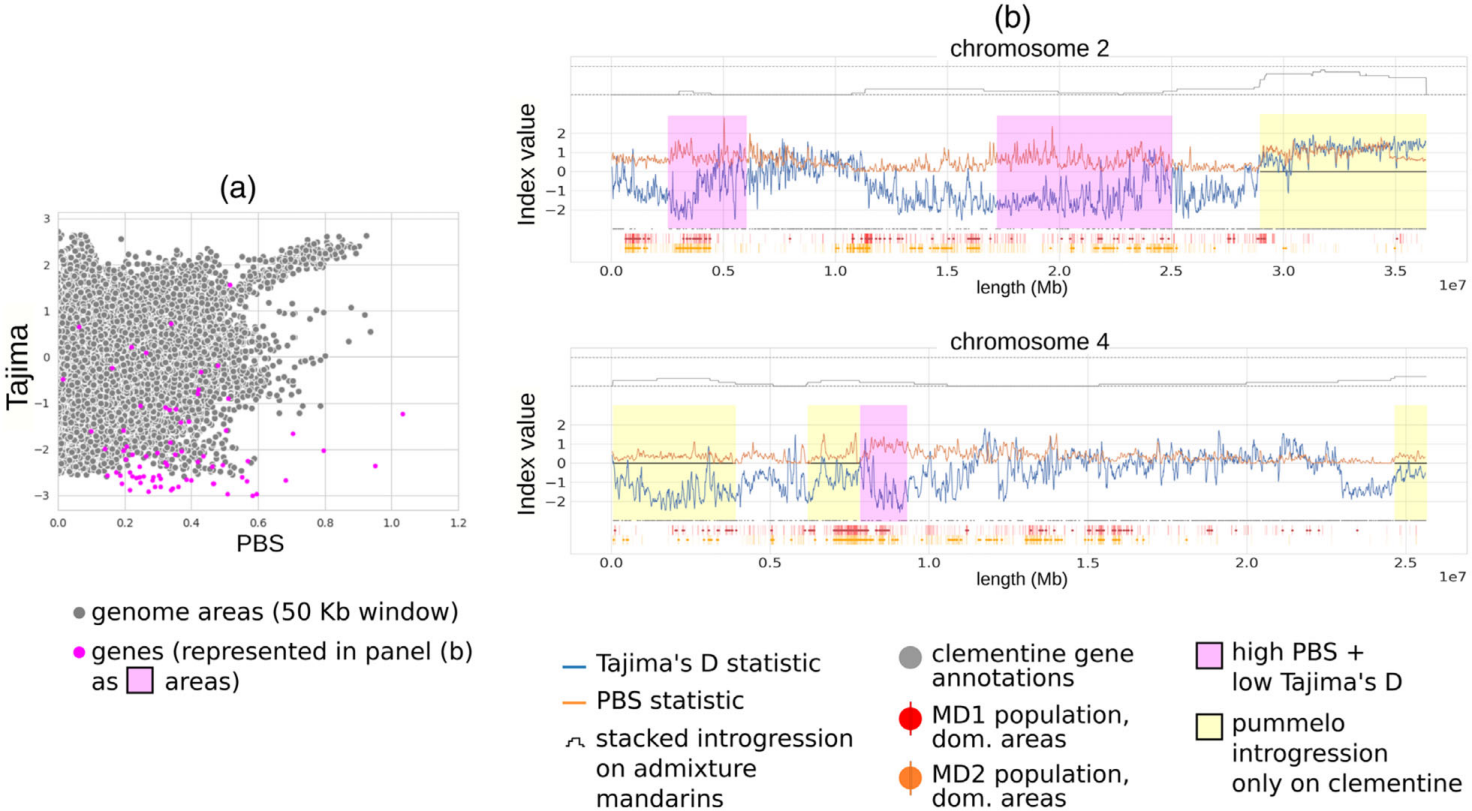
Population genomic analysis of several wild and cultivated mandarins (*Citrus reticulata*). Population Branch Statistic (PBS) and Tajima's D statistics were calculated per 50 Kb windows of genome sequence. (a) Scatter plot of Tajima's D vs PBS statistics. Index values were calculated for the genome sequence (grey) and for genes (pink). (b) Line plot of Tajima's D and PBS values represented on the clementine genome sequence of chromosomes 2 and 4. Clementine genome MD1 and MD2 regions, the two domestication areas identified elsewhere on *Citrus sinensis* (Wang et al., 2018), are represented by red and orange bars, respectively. Bars with a dot represent areas longer than 1,000 nt. Annotated genes of clementine are shown in grey dots as a reference. Histogram of stacked introgressed pummelo areas in admixture mandarins (Wu et al., 2018) is shown on the top of the figure

Underlying the chromosomal level, Tajima's D of both pummelo and mandarin regions also revealed spots of opposite trends. Introgression can be related to high positive or negative determinations, as for instance at the beginning of chromosome 4 and the end of chromosome 2 (Figure 1b). It is worth mentioning that the gene responsible for nucellar embryony in citrus (Wang et al., 2017), a well‐known beneficial agricultural trait that allows asexual propagation (Rao et al., 2021), was located in a broad area with negative Tajima's values (chromosome 1, coordinates 23–26 Mb; Supplemental Figure S2), reflecting selective pressure against changes in this population of mandarins to conserve this trait. Crossing our results with genome regions of orange, namely MD1 and MD2, potentially related to mandarin domestication (Wang et al., 2018) reduced to 183 the minimum core of genes (Supplemental Table S1). They included a MATE efflux family protein, a cation/H+ exchanger and a vacuolar ATPase that is highly expressed in fruit (Terol et al., 2016). Genome areas where low negative Tajima's (D less than −1) and high positive PBS (>0.3) values converge are relevant (Figure 1a) because they shed light on the highest dissimilarities that have been retained in Type III cultivars. Interestingly, these values were concentrated in two regions on chromosomes 2 and 4 (Figure 1b, pink areas) that shared 75 genes, including several strictosidine synthases, involved in the synthesis of monoterpenoid indole alkaloids.

Despite the general trends, on every chromosome there were peaks of negative Tajima's D values in an otherwise positive background, pointing to a few or even single candidate genes as major players of the effect of pummelo. To take into account the effect at the gene level, we calculated both indexes per coding region; genes were ranked according to their values, and their functional annotation was analyzed. Detected GO categories (Supplemental Table S2) included pH regulation (GO:0006885) (mostly cation/H+ exchangers, four of which are potentially vacuolar proteins) and hydrogen ion transmembrane transport (GO:1902600). Mitochondrial processes were also well represented with two additional categories, tricarboxylic acid cycle (TCA; GO:0006099), including phosphoenolpyruvate carboxykinase, succinate dehydrogenase, isocitrate lyase, citrate synthase, and 2‐oxoglutarate dehydrogenase, all of which are expressed in citrus fruit (Terol et al., 2016), and mitochondrial transport proteins (GO:0006839).

Genes ranked according to their PBS values revealed different overrepresented GO categories (Supplemental Table S3). The most enriched GO term was related to transcription regulation with hundred of entries including response to ethylene (AP2 domain, PF00847), B3 domain (PF02362), WRKY (PF03106), and Myb (PF00249, PF13837, PF13921) transcription factors, homeobox genes (PF00046, PF05920, PF02183), and auxin‐responsive elements (PF02309, PF06507). This observation supports that transcription factors underlie many domestication traits (Doebley et al., 2006). A vast number of diverse methyltransferases were also detected, highlighting again the importance of such proteins in citrus (see companion publication Gonzalez‐Ibeas et al., 2021). Almost 200 genes related to defense response were also featured by high PBS indexes. Photosynthesis acclimation was also found to differ between cultivated and wild mandarins. Several genes associated with arsenic‐responsive membrane transporters, gene silencing (a *DCL2*), acidity and the TCA) among others were also identified.

### 2.9 Contribution of pummelo introgression to fruit size

**FIGURE 2:**
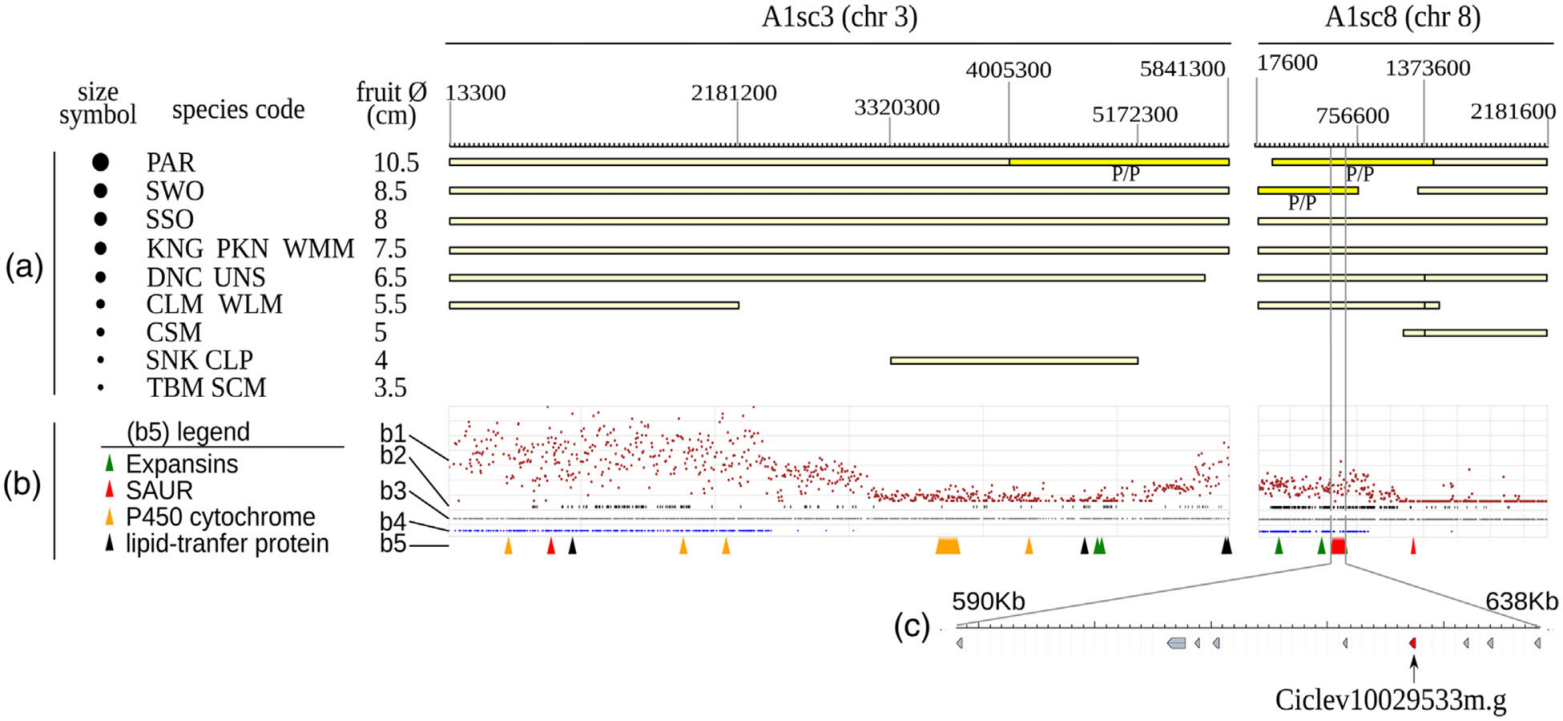
Contribution of pummelo introgression to fruit size in mandarin genomes. (a) Overlapping pummelo introgressed areas on chromosomes 3 and 8 in citrus admixtures including mandarin (*Citrus x reticulata*), orange [*Citrus x sinensis* (L) Osbeck], and grapefruit (*C. x paradisi* Macfadyen), with different fruit diameter (cm). Heterozygous introgression = light yellow (P/M); homozygous introgression = dark yellow (P/P). Genome coordinates (nt) of major breaking points are shown on the top of introgression bars. (b) Genomic features (from top to bottom): (b1) PBS indexes (brown dots); (b2) SNP positions (black bars) contributing to principal component P1 of PCA analysis (Figure 3); (b3) genes annotated (grey) on the clementine genome as reference for estimation of gene density background; (b4) SNP positions (blue) with alleles showing correlation with fruit size; (b5) selected genes with functional annotation potentially related to cell fruit size. (c) Cluster of genes annotated as small auxin‐responsive factors (SAURs) on A1sc8 area. Gene Ciclev10029533m.g is highlighted in red, and expanded nucleotide and protein sequence analysis is provided in Supplemental Figure S3. Species codes as described in Supplemental Table S5

It has been reported that fruit size in domesticated varieties correlates with the proportion of introgressed pummelo (Wu et al., 2018), but the genetic determinants responsible of this effect have not been elucidated. To investigate this issue, we stacked introgression areas, which identified two fragments on chromosomes 3 (5.8 Mb) and 8 (2.2 Mb), named A1sc3 and A1sc8, respectively (Figure 2), whose introgressed pummelo fragments correlated to mandarin fruit size. Furthermore, the larger fruit sizes of sweet orange [*Citrus × sinensis* (L.) Osbeck] and grapefruit (*C. x paradisi* Macfadyen) are apparently related to a second pummelo introgression leading to homozygous regions (Figure 2). These two regions encompassed 797 genes (chromosome 3) and 327 genes (chromosome 8), some of which can be found in tandem in large clusters. This is suggestive of extra doses of genes that potentially regulate fruit size based on their functional annotations, including cell growth and expansion (nine auxin responsive genes [*SAUR*] and 6 expansins; Stern et al., 2007; Devoghalaere et al., 2012), phytohormone biosynthesis (14 cytochromes P450; Bak et al., 2011), or cuticle formation (seven lipid‐transfer proteins).

**FIGURE 3:**
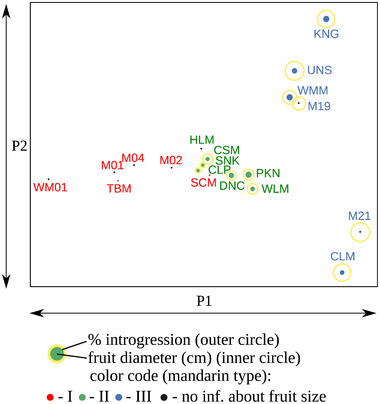
Principal component analysis (PCA) of allele frequencies calculated from single nucleotide polymorphism (SNP) data by PCAdapt in pure (Type I) and admixture (Type II and III) mandarins (*Citrus x reticulata*) colored in red, green and blue, respectively. Sample metadata include percentage of introgressed pummelo in Mb (yellow outer circumference) ranging from 0.3% (CLP) to 22.9% (UNS), and fruit diameter in cm (colored inner circle) ranging from 3 cm (CLP) to 7.5 cm (PKN). Black inner circle denotes no information about fruit size. For species code see Supplemental Table S5

We carried out PCA on SNP allele frequencies among Type I, II, and III mandarins (Figure 3). PCAdapt was used to recover SNPs linked to the horizontal component (P1), the best one describing variation in fruit size. In total, 792 and 3,252 SNPs located in 124 and 153 genes were identified on A1sc3 and A1sc8 areas, respectively (Figure 2b, black dots). It is worth mentioning that the highest concentration of SNPs was found in the chromosome 8 fragment, despite being smaller, and also that these SNPs overlapped with the double introgression present in sweet orange. Since P1 best describes the fruit size shift but may also represent other sources of biological variation, we manually examined SNP patterns among citrus samples with any correlation to fruit size (e.g., gene Ciclev10029533m.g, the only member of the SAUR cluster that met these criteria) (Supplemental Figure S3). Exploration of the whole genome showed that such pattern was highly skewed to the onset of chromosomes 3 and 8 (Figure S3B and Figure 2b4, blue dots), where 3,864 and 588 SNPs were found, respectively, overlapping with 231 and 85 genes leading to at least one amino acid change (Supplemental Table S4). Among the full list of candidates, Ciclev10029533m.g appears to have some relevance as a regulator of fruit size since it has similarity to *Arabidopsis SAUR64*, which is highly expressed in developing siliques (http://travadb.org/). Some *SAUR* genes induce cell elongation through cell wall acidification after interaction with PP2C phosphatases and phosphorylation of H(+)‐ATPases (Spartz et al., 2014). PP2C Phosphatase (*Ciclev10028600m.g*) and H(+)‐ATPase 4 (*Ciclev10018737m.g*) displaying the same zygosity pattern that the *SAUR*‐like gene were also detected within A1sc3 and A1sc8 areas as candidates (Supplemental Table S4). These regions also included expansins, F‐box proteins and one auxin response factor, gene types reported to participate in fruit size control in other crops (Devoghalaere et al., 2012; de Jong et al., 2015). From an evolutionary standpoint, it is worth mentioning that the three nucleotide positions in *Ciclev10029533m.g* correlated to fruit size were also identified under selective pressure by NSsites analysis (yellow arrows in Supplemental Figure S3A1) in the companion publication (Gonzalez‐Ibeas et al., 2021). The A1sc3 and A1sc8 areas contained 24 additional genes with SNPs correlated to fruit size which underwent positive selection during evolution (Supplemental Table S4), including transcription factors and membrane transporters. Moreover, genes derived from previously discovered ancient whole genome duplications (WGD) (Gonzalez‐Ibeas et al., 2021) were also identified in these areas as candidates of fruit size determination (Supplemental Table S4). In chromosome 3, for instance, one member of a pectin lyases WGD triplet, *Ciclev10019941m.g*, was found. We manually confirmed the episodic adaptive selection in the pummelo lineage of this gene that was detected by a free‐ratio PAML test (Gonzalez‐Ibeas et al., 2021) (Supplemental Figure S4).

**FIGURE 4:**
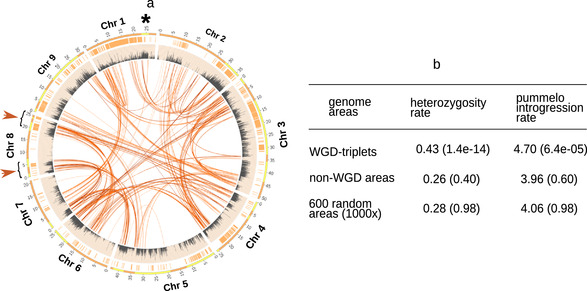
(a) Circos diagram of the clementine (*Citrus clementina*) genome, the reference genome for citrus (Wu et al., 2014). Each ideogram represents a chromosome where introgressed pummelo areas are highlighted in yellow and pure mandarin areas in orange color. Track 1 depicts areas in orange color with recent admixture proportion among mandarins identified by NGSadmix. Track 2 shows a histogram of the level of heterozygosity in the genome. Ribbons connect duplicated areas (syntelogs) derived from the paleohexaploidy event shared by eudicots (see companion publication Gonzalez‐Ibeas et al., 2021). (b) Heterozygosity and pummelo introgression rates of several genome areas. The significance of the difference of means between each data set and the entire genome (analyzed in 10 Kb nonoverlapping windows) was tested by one‐sample *t*‐test. *P*‐values are shown in parentheses. In addition, 500 nonoverlapping genome areas of 10 Kb in length were chosen randomly and heterozygosity and introgression rates were compared with the genome background value by the same test. The analysis was repeated 1,000 times. Row number 3 shows average results after *P*‐value correction for multiple testing

Intriguingly, there is prominent overlap of gene triplets from WGD‐derived areas with hotspots of heterozygosity (e.g., chromosome 8, Figure 4a, brown arrows). Indeed, the heterozygosity rate of these areas was significantly higher than that of the entire genome (Figure 4b). To determine whether this could occur by chance, we randomly sampled 600 genome regions of similar length to WGD‐derived triplets repeated a thousand times, which resulted in no statistical significance (Figure 4b). The data also indicated that only a fraction of these heterozygous areas derived from pummelo introgression. Heterozygosity of pummelo areas on mandarin genomes has previously been attributed to the effect of introgression (Wu et al., 2018), but mandarin domestication has also involved a complex network of back crosses among several mandarin cultivars (Wu et al., 2018; Wang et al., 2018; Talon et al., 2020). The latter could explain the additional heterozygosity outside pummelo introgressions. To verify this hypothesis, recent admixture proportions of clementine with each of the analyzed mandarins were estimated with NGSadmix. Many spots of heterozygosity with no pummelo contribution could in fact be explained by admixture among mandarins (Figure 4a); for example, in chromosome 1 (coordinates 1–20 Mb), chromosome 2 (coordinates 6–12 Mb), chromosome 3 (coordinates 2–8 Mb), chromosome 4 (coordinates 13–23 Mb), among others. Thus, this link between heterozygosity and introgressions suggests that WGD syntelogs may be recurrently targeted by successive domestication steps.

## 3 DISCUSSION

In this work, we present a study on the major genomic determinants of domestication of the citrus gene space. Pathogen‐defense genes are recurrent targets of evolution in many genera (Zhang et al., 2019), and we have shown that citrus is not an exception (Gonzalez‐Ibeas et al., 2021). Domestication also affected these genes, since a statistically significant proportion were identified with high PBS values, indicating divergence of cultivars from wild relatives. Conversely, no significance was found for negative Tajima's D values, suggesting that pest resistance is not a primary target of domestication (pest control can involve cultural practices and external treatments). Alternatively, there may be other explanations for the increased Tajima's D values, such as equal distribution of common alleles (balancing selection), which is a well‐accepted phenomenon for pathogen‐resistance genes (Fijarczyk & Babik, 2015). It is also widely accepted that crop domestication may lead to a decrease in disease or insect/pest resistance in cultivars compared with wild relatives, also reported for citrus (Rao et al., 2021). Higher yields and better flavor or scent at the expense of weaker defense against pests can be achieved via reduction of such chemical defenses as phenolics, cyanogenic glycosides, glucosinolates, and alkaloids, which are generally distasteful (Matsuura & Fett‐Neto, 2015). An example is domesticated cabbage (*Brassica oleracea* L.), which contains lower levels of glucosinolates than its wild relatives (Moreira et al., 2018). In our study, citrus strictosidine synthases, which play a role in the indole‐alkaloid pathways, were identified in genome areas with low negative Tajima and high positive PBS values, denoting both retention during domestication and divergence from wild relatives. Some of these enzymes were expressed in flower or root but not in fruit (Terol et al., 2016), providing a potential example of tissue‐targeted reduction of unpalatable compounds (Whitehead et al., 2017). Similarly, a mandelonitrile lyase was found in the lineage‐specific gene content of unpalatable citron and ‘Sun Chu Sha’ mandarin but absent in edible clementine (Gonzalez‐Ibeas et al., 2021). These enzymes are involved in the release of hydrogen cyanide from cyanogenic glycosides, a toxic compound for human intake but involved in pathogen defense (Bolarinwa et al., 2016).

Another pivotal component of fruit flavor is acidity. Fruit of nonedible wild citrus species are generally very acidic and the reduction of citric acid in their vesicles appears to be a critical trait for domestication. It has been proposed (Rao et al., 2021) that several mutations in the regulatory transcription factor module controlling acidity accumulation are responsible of this trait (Butelli et al., 2019). Moreover, other biosynthetic and metabolic genes have also been suggested to contribute to citric acid homeostasis in citrus, such as isocitrate dehydrogenase (Wu et al., 2018) or aconitase (Wang el al., 2018). Our data show that GO categories related to acidity regulation are enriched in chromosome regions with negative Tajima values, although no statistical significance was found for PBS values. This observation indicates that the metabolic machinery regulating acidity (mainly the TCA cycle, cation/H+ exchangers, vacuolar proteins, and hydrogen ion transmembrane transporters) has generally been conserved during mandarin domestication and that the changes in acidity were probably achieved modifying specific regulatory elements via ancient mutations, as suggested (Wang et al., 2018).

Pummelo introgression into the ancestral mandarin genome is thought to constitute an important element of the domestication history of citrus (Talon et al., 2020). The onset of chromosomes 3 and 8 are very likely main drivers of these processes since they are shared by almost every cultivated variety. We hypothesize that these fragments may contribute to the regulation of fruit size, although our results and the complexity of this quantitative trait appears to suggest a cooperative effect of several genetic determinants, as described for example in Solanaceae (Wang et al., 2015). However, key elements identified here (e.g., the SAUR gene *Ciclev10029533m.g* and the pectin lyase *Ciclev10019941m.g*) are candidates for fruit size regulation, and both connect domestication to previous evolutionary events, the former through pervasive adaptation, and the latter through an episodic differentiation in pummelo (Gonzalez‐Ibeas et al., 2021) that was incorporated later into mandarin by introgression. As mentioned above for acidity, we also identified nearly pure mandarin areas with negative Tajima's indexes, low pummelo introgression rates and low PBS values, contributing to domestication by retaining key mandarin traits, such as nucellar embryony that allowed considerable expansion of desired genotypes, where foreign introgression was likely restricted. In maize, for example, resistance to wild introgression has been described for regions containing several domestication genes (Hufford et al., 2013). In line with this, there is a lower rate of admixture among mandarins at the end of chromosome 1 where the nucellar embryony *locus* is embedded, compared with the rest of the chromosome. In addition to pummelo introgression, further improvements along domestication arose from new crosses between ancestral hybrids and admixtures, giving rise to the current basic types of edible fruits. This process has etched cultivated citrus genomes with spots of heterozygosity consequence of introgressions that reveal genome areas repeatedly targeted during selection. Much has been speculated about the frequent introgressions from wild relatives into several crop varieties and their role in, for example, local adaptation (Janzen et al., 2019). Our results suggest a significant prevalence of WGD‐derived areas overlapping with these spots that may be due to the same principle as for wild introgression, that is, ancient genetic material that was important for founding the species in the past through natural selection could later be exploited during artificial selection, highlighting potential genes of interest to be investigated in the future for breeding. The pectin lyase *Ciclev10019941m.g* represents a good example again. It combines paleohexaploidy origin, episodic adaptation, introgression, and SNP pattern correlating with a trait of agricultural interest.

In conclusion, our results show that citrus domestication, as in other crops, mostly affected gene families related to transcription regulators. Palatability is a main target since we identified genes involved in biogenesis of distasteful compounds and regulating pH balance and citric acid metabolism. The data also suggest that pummelo introgressions contributed to the increase in ancestral mandarin size. Other key genes of citrus domestication were located in conserved stretches of pure mandarin regions, such as the putative apomictic reproduction gene that allowed huge dispersion of the desirable domesticated genotypes of citrus. Finally, we propose that in this genus, as a long‐lived perennial with limited breeding opportunities, certain genes that were the subject of natural selection or duplication later became potential targets of domestication, a clear‐cut difference with annual plants, that could likely target a wide range of genes through many rounds of domestication.

## AUTHOR CONTRIBUTIONS

Daniel Gonzalez‐Ibeas: Conceptualization, Formal analysis, Investigation, Methodology, Software, Visualization, Writing‐original draft, Writing‐review & editing. Victoria Ibanez: Project administration, Validation. Estela Perez‐Roman: Data curation, Validation, Writing‐review & editing. Carles BorredÃ¡: Data curation, Software, Validation, Writing‐review & editing. Javier Terol: Funding acquisition, Investigation, Project administration, Software, Supervision, Writing‐review & editing. Manuel Talon: Conceptualization, Funding acquisition, Investigation, Project administration, Supervision, Writing‐original draft, Writing‐review & editing.

## CONFLICT OF INTEREST

The authors declare no conflict of interest.

## Supporting information

Figure S1: Tajims's D and PBS index analysis
Figure S2: Line plots of Tajima's D and PBS values across citrus chromosomes
Figure S3: Multiple alignment of protein sequences and SNP analysis of SAUR gene Ciclev10029533m.g.
Figure S4: Multiple alignment of protein sequences and SNP analysis of pectin lyase gene Ciclev10019941m.g
Table S1. Tajima's D values calculated for genes overlapping with areas involved in mandarin domestication identified in *Citrus sinensis*.
Table S2. Gene Ontology categories of genes with low negative Tajima's D values
Table S3. Gene Ontology categories of genes with high PBS values

Table S4. Genes on pummelo introgressed areas potentially involved in citrus fruit size control

Table S5. Sample description and GenBank accessions
