# Supplementary material for: Shaping the biology of citrus: II. Genomic determinants of domestication
Source: Plant Genome. 2021 Aug 31;14(3):e20133. doi: 10.1002/tpg2.20133 (PMC12807309; doi:10.1002/tpg2.20133)
Supplement: Supplementary file 1 [file TPG2-14-e20133-s003.pdf]

Running title: Genomics of citrus domestication

Shaping the Biology of Citrus II: Genomic Determinants of Domestication

Daniel Gonzalez-Ibeas, Victoria Ibanez, Estela Perez-Roman, Carles Borredá, Javier Terol, Manuel Talon*

Affiliations: Valencian Institute of Agricultural Research (IVIA). Carretera Moncada CV-315, Km 10, 46113, Valencia, Spain.

*corresponding author: talon_man@gva.es

Supplementary figures.

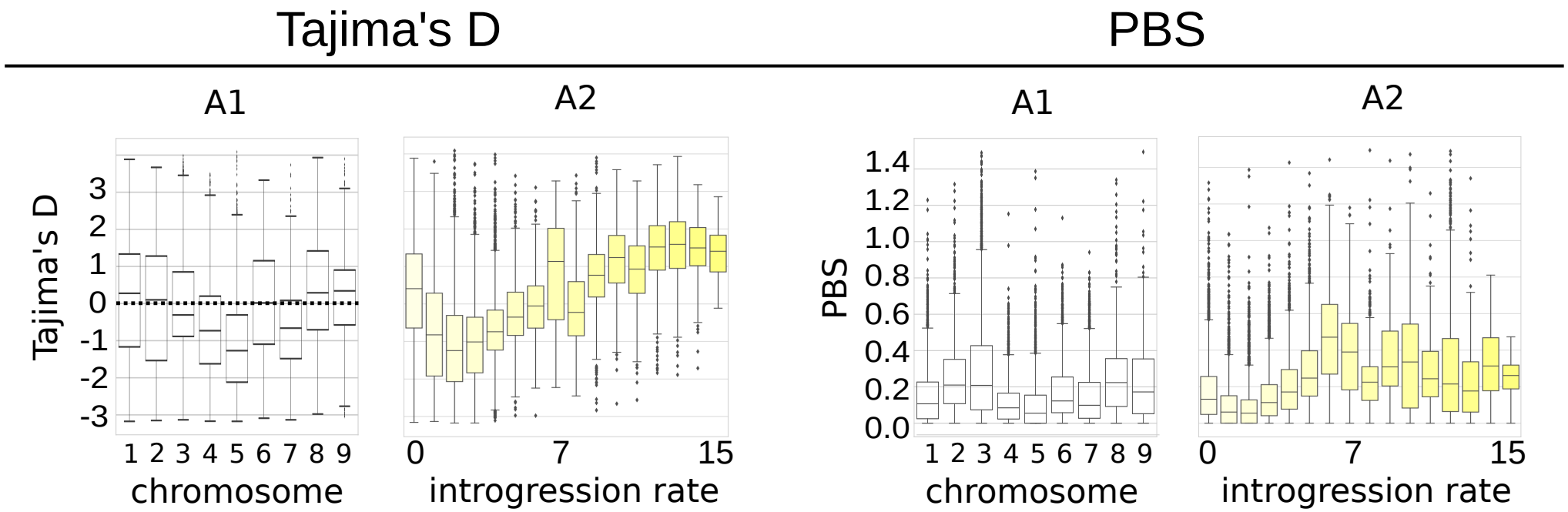

Figure S1. Box plots of Tajima's D and PBS values grouped (A1) per chromosome, (A2) per introgression rate, measured as the number of times a pummelo introgression has been identified in any mandarin accession.

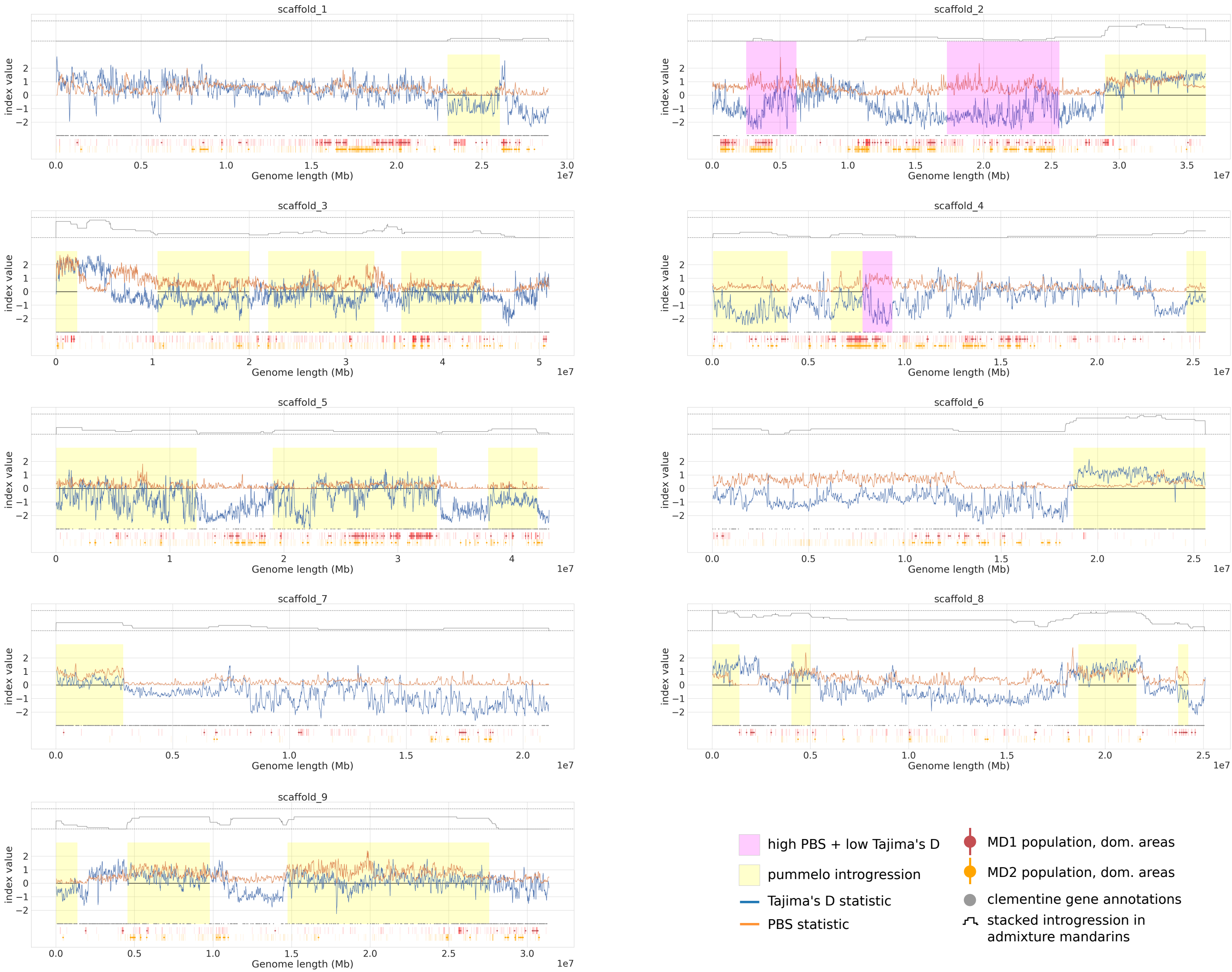

Figure S2. Line plots of Tajima's D and PBS values across all chromosomes of the clementine genome sequence. Clementine genome MD1 and MD2 regions, the two domestication areas identified elsewhere on Citrus sinensis (Wang et al. 2018), are represented by red and orange bars, respectively. Bars with a dot represent areas longer than 1000 nt. Annotated genes of clementine are shown in grey dots as a reference. Histogram of stacked introgressed pummelo areas (i.e. introgression rate, measured as the number of times a pummelo introgression has been identified in any admixture mandarin, Wu et al., 2018) is shown on the top of the figure.

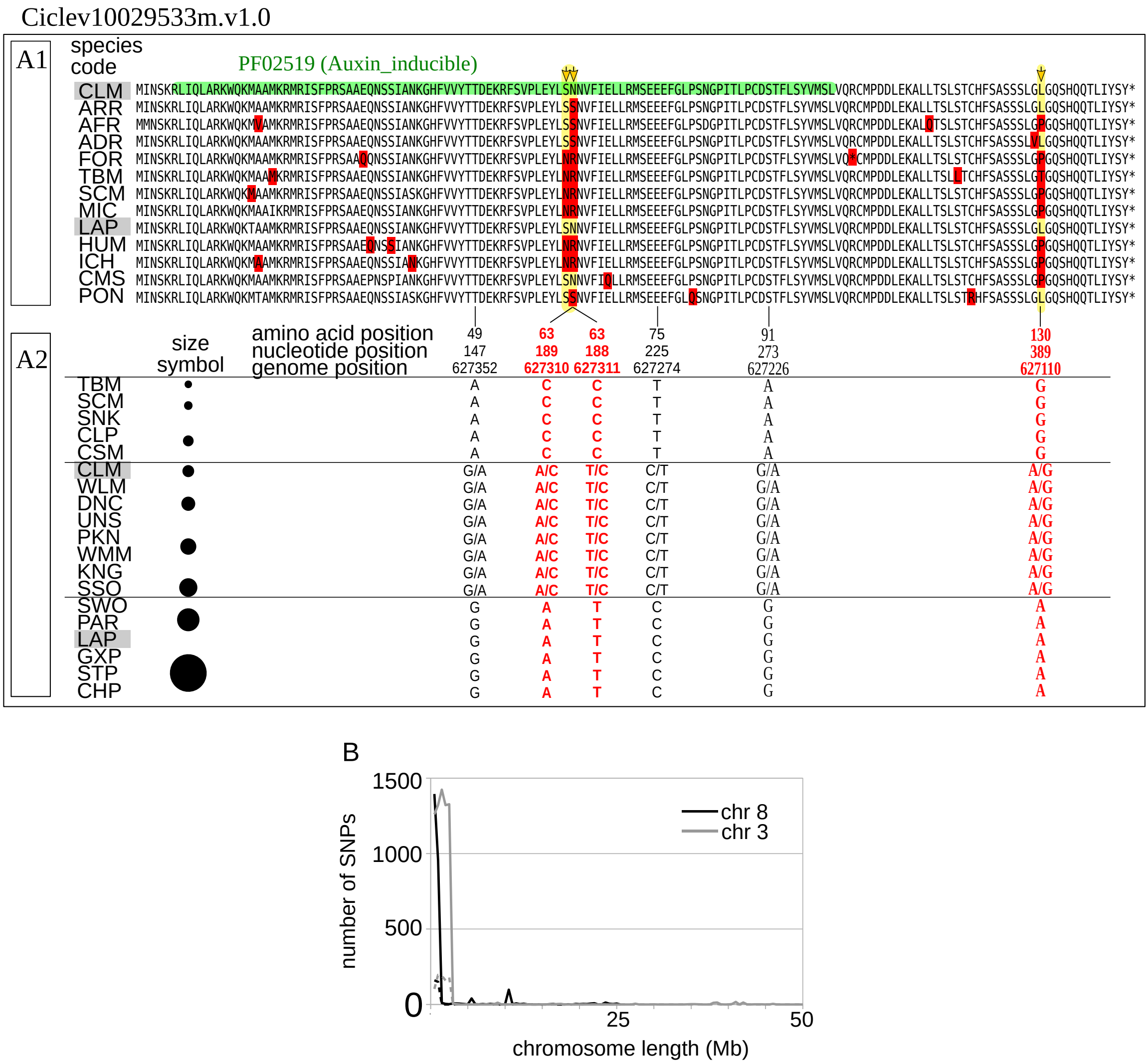

Figure S3. (A) Multiple alignment of protein sequences of gene *Ciclev10029533m.g* in citrus species (A1) and SNP analysis in citrus admixtures of different fruit diameter (A2). Green bar represents the PF02519 protein domain of SAUR genes. Non-synonymous nucleotide changes are in red color. Amino acid changes relative to the clementine reference genome are highlighted in red too. Yellow arrows point amino acid positions under positive selective pressure during citrus evolution, identified by the NSsites analysis with PAML (see companion publication Gonzalez-Ibeas et al., 2021). (B) Number of SNP positions correlating with fruit size along the entire sequence of chromosomes 3 and 8 calculated per windows of 200 Kb.

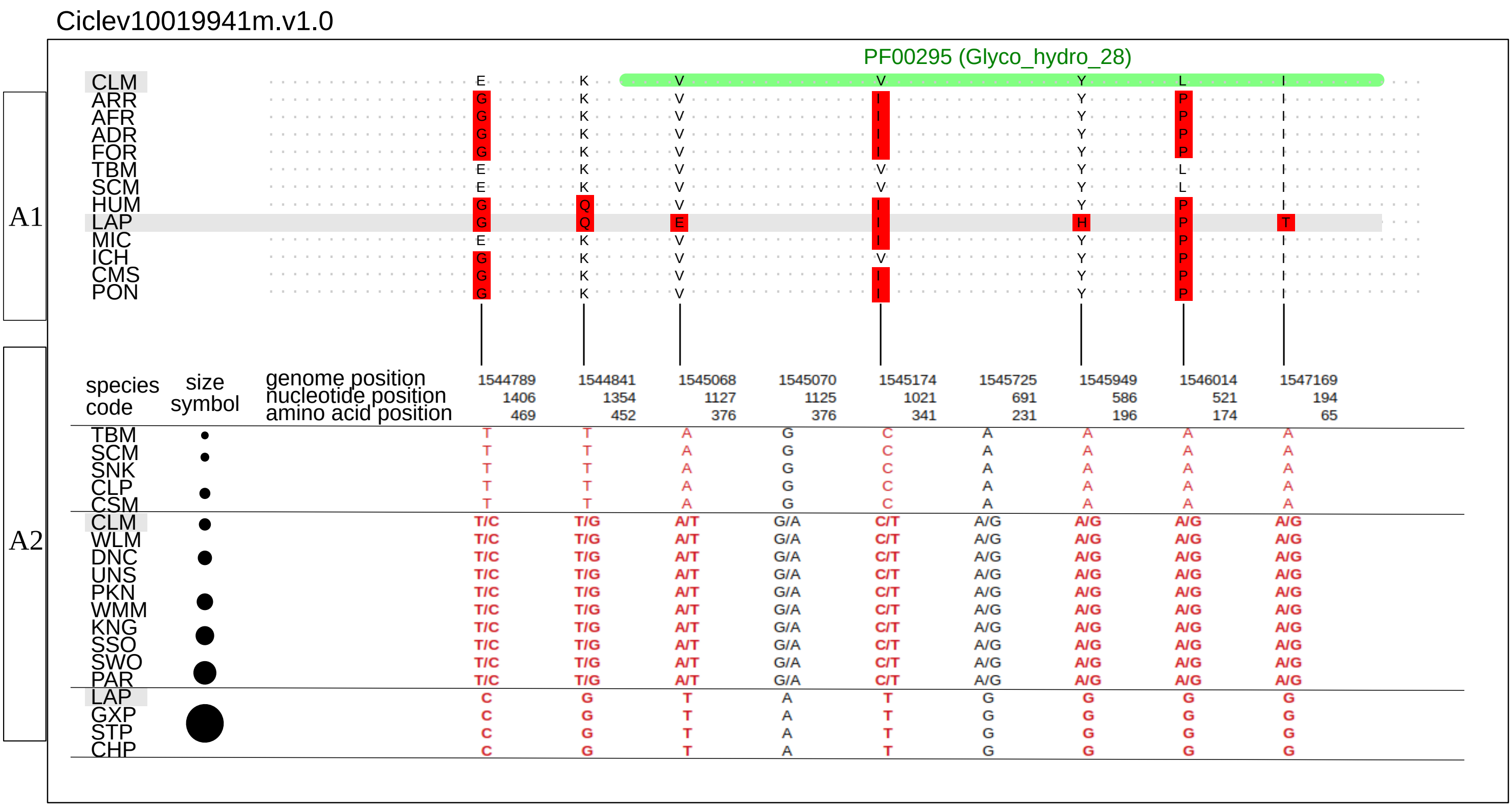

Figure S4. Protein (A1) and nucleotide (A2) changes as related to the clementine reference genome in the pectin lyase gene *Ciclev10019941m.g* identified from multiple alignment of protein sequences in pure citrus species (A1) and SNP analysis in citrus accessions of different fruit diameter (A2). Only changes are represented for each sequence. In A1, green bar represents the PF00295 (Glycoside hydrolase family 28) protein domain. In A2, non-synonymous and synonymous nucleotide substitutions are highlighted in red and grey, respectively. Clementine (CLM) and pummelo (LAP) data are highlighted in grey boxes. LAP accumulated 3 unique amino acid changes compared to other pure wild species, which was manifested in a markedly higher free-ratios-omega value in pummelo during citrus evolution (see companion publication Gonzalez-Ibeas et al., 2021). Species codes see Table S7.

Supplementary Tables.

Table S1. Tajima's D value calculated per gene sequence for genes whose value was lower than 1 and that overlapped with MD1 and MD2 areas, the two domestication areas identified elsewhere on Citrus sinensis (Wang et al. 2018)

Gene ID	Tajima's D	Functional annotation
Ciclev10000638m.g	-3.099125	Tetratricopeptide repeat (TPR)-like superfamily protein
Ciclev10033495m.g	-3.071363	GDSL-like Lipase/Acylhydrolase superfamily protein
Ciclev10003268m.g	-2.99039	Ankyrin repeat family protein
Ciclev10016308m.g	-2.960993	Ribosomal protein L2 family
Ciclev10017934m.g	-2.957757	no_ann
Ciclev10017908m.g	-2.899021	Peroxidase superfamily protein
Ciclev10002637m.g	-2.865991	E2F/DP family winged-helix DNA-binding domain
Ciclev10033408m.g	-2.835894	no_ann
Ciclev10031477m.g	-2.775606	Single-stranded nucleic acid binding R3H protein
Ciclev10003374m.g	-2.769126	Ankyrin repeat family protein
Ciclev10031166m.g	-2.759688	Zn-dependent exopeptidases superfamily protein
Ciclev10027409m.g	-2.759486	no_ann
Ciclev10000243m.g	-2.754581	extra-large GTP-binding protein 3
Ciclev10003438m.g	-2.739304	Protein kinase superfamily protein
Ciclev10018065m.g	-2.731782	Tetratricopeptide repeat (TPR)-like superfamily protein
Ciclev10000200m.g	-2.728621	Glycosyl hydrolases family 31 protein
Ciclev10013013m.g	-2.711363	no_ann
Ciclev10031999m.g	-2.698826	Radical SAM superfamily protein
Ciclev10003360m.g	-2.675273	Ankyrin repeat family protein
Ciclev10013135m.g	-2.674002	Bifunctional inhibitor/lipid-transfer protein/seed storage 2S albumin superfamily protein
Ciclev10002389m.g	-2.651131	SNF7 family protein
Ciclev10027266m.g	-2.624326	UDP-Glycosyltransferase superfamily protein
Ciclev10032630m.g	-2.613444	Protein of unknown function (DUF674)
Ciclev10033622m.g	-2.557291	heat shock cognate protein 70-1
Ciclev10016580m.g	-2.54791	Cysteine proteinases superfamily protein
Ciclev10003273m.g	-2.526046	NAC domain containing protein 102
Ciclev10001364m.g	-2.509907	Galactosyltransferase family protein
Ciclev10023431m.g	-2.495673	NmrA-like negative transcriptional regulator family protein
Ciclev10018247m.g	-2.483287	no_ann
Ciclev10000505m.g	-2.476928	no_ann
Ciclev10001736m.g	-2.470092	Pseudouridine synthase family protein
Ciclev10015460m.g	-2.462193	mitogen-activated protein kinase kinase kinase 18
Ciclev10000923m.g	-2.461469	GHMP kinase family protein
Ciclev10015816m.g	-2.442201	pyrimidin 4
Ciclev10007337m.g	-2.42043	Clp amino terminal domain-containing protein
Ciclev10008675m.g	-2.412885	Afadin/alpha-actinin-binding protein
Ciclev10003287m.g	-2.382882	Ankyrin repeat family protein
Ciclev10026032m.g	-2.37667	Integrase-type DNA-binding superfamily protein
Ciclev10017942m.g	-2.359463	MATE efflux family protein
Ciclev10017882m.g	-2.332063	P-loop containing nucleoside triphosphate hydrolases superfamily protein
Ciclev10002744m.g	-2.331582	Protein of unknown function (DUF59)
Ciclev10024790m.g	-2.297711	Domain of unknown function (DUF1726) ;Putative ATPase (DUF699)
Ciclev10007391m.g	-2.276761	no_ann

Ciclev10002914m.g	-2.270608	ssDNA-binding transcriptional regulator
Ciclev10014459m.g	-2.249981	tetratricopeptide repeat (TPR)-containing protein
Ciclev10017358m.g	-2.239763	UDP-Glycosyltransferase superfamily protein
Ciclev10015281m.g	-2.231482	eukaryotic release factor 1-3
Ciclev10000085m.g	-2.228863	phosphatidylinositol 4-OH kinase beta1
Ciclev10002024m.g	-2.222876	adenine phosphoribosyl transferase 1
Ciclev10007313m.g	-2.220582	lysine-ketoglutarate reductase/saccharopine dehydrogenase bifunctional enzyme
Ciclev10017763m.g	-2.220117	no_ann
Ciclev10000803m.g	-2.199014	Transcription elongation factor (TFIIS) family protein
Ciclev10027252m.g	-2.189902	ribonucleotide reductase 1
Ciclev10016257m.g	-2.150509	RING/U-box superfamily protein
Ciclev10032567m.g	-2.144173	Protein of unknown function (DUF674)
Ciclev10014674m.g	-2.144056	Tetratricopeptide repeat (TPR)-like superfamily protein
Ciclev10032854m.g	-2.129365	zinc ion binding;nucleic acid binding
Ciclev10015126m.g	-2.126428	O-acyltransferase (WSD1-like) family protein
Ciclev10001875m.g	-2.122795	no_ann
Ciclev10018036m.g	-2.103648	FAD/NAD(P)-binding oxidoreductase family protein
Ciclev10017356m.g	-2.103595	no_ann
Ciclev10012710m.g	-2.101622	glutathione S-transferase TAU 8
Ciclev10003669m.g	-2.094789	SH3 domain-containing protein
Ciclev10033468m.g	-2.076345	no_ann
Ciclev10024904m.g	-2.074908	STELAR K+ outward rectifier
Ciclev10033479m.g	-2.064031	Major facilitator superfamily protein
Ciclev10002859m.g	-2.062526	thioredoxin H-type 1
Ciclev10015347m.g	-2.057932	aspartate aminotransferase 1
Ciclev10001187m.g	-2.054258	DC1 domain-containing protein
Ciclev10024645m.g	-2.048863	no_ann
Ciclev10000393m.g	-2.045319	beta-galactosidase 10
Ciclev10000897m.g	-2.0438	calcium-dependent protein kinase 29
Ciclev10017229m.g	-1.992671	Bifunctional inhibitor/lipid-transfer protein/seed storage 2S albumin superfamily protein
Ciclev10002564m.g	-1.933522	adenine phosphoribosyl transferase 1
Ciclev10032050m.g	-1.917075	binding to TOMV RNA 1L (long form)
Ciclev10018380m.g	-1.88981	Ras-related small GTP-binding family protein
Ciclev10001136m.g	-1.883848	Regulator of chromosome condensation (RCC1) family protein
Ciclev10014134m.g	-1.868914	ARM repeat superfamily protein
Ciclev10017909m.g	-1.862077	Peroxidase superfamily protein
Ciclev10001163m.g	-1.859139	Translation initiation factor IF2/IF5
Ciclev10031853m.g	-1.857006	dual specificity protein phosphatase (DsPTP1) family protein
Ciclev10033206m.g	-1.847683	no_ann
Ciclev10000986m.g	-1.842381	amino acid permease 2
Ciclev10033480m.g	-1.840579	Serine carboxypeptidase S28 family protein
Ciclev10032023m.g	-1.835801	Protein of unknown function DUF455
Ciclev10031520m.g	-1.833327	P-loop containing nucleoside triphosphate hydrolases superfamily protein
Ciclev10007283m.g	-1.823132	DNA ligase IV
Ciclev10000420m.g	-1.8127	Glycosyl hydrolase family 35 protein
Ciclev10015554m.g	-1.796583	O-acyltransferase (WSD1-like) family protein
Ciclev10014057m.g	-1.790436	no_ann
Ciclev10002943m.g	-1.785548	no_ann
Ciclev10009588m.g	-1.778262	HSP20-like chaperones superfamily protein

Ciclev10030714m.g	-1.764603	hercules receptor kinase 1
Ciclev10033260m.g	-1.727518	no_ann
Ciclev10015375m.g	-1.726689	SHAGGY-related protein kinase dZeta
Ciclev10025412m.g	-1.725812	cytochrome P450, family 96, subfamily A, polypeptide 10
Ciclev10033003m.g	-1.722279	no_ann
Ciclev10015269m.g	-1.695288	heat shock transcription factor A1D
Ciclev10030480m.g	-1.687188	kow domain-containing transcription factor 1
Ciclev10016493m.g	-1.684679	early nodulin-like protein 1
Ciclev10016047m.g	-1.676059	bZIP protein
Ciclev10018119m.g	-1.675581	NADH:cytochrome B5 reductase 1
Ciclev10014800m.g	-1.674788	Transducin/WD40 repeat-like superfamily protein
Ciclev10030746m.g	-1.674081	Leucine-rich repeat receptor-like protein kinase family protein
Ciclev10017680m.g	-1.673343	sulphotransferase 12
Ciclev10018047m.g	-1.671632	laccase 17
Ciclev10007535m.g	-1.644839	P450 reductase 2
Ciclev10030969m.g	-1.644206	vacuolar ATP synthase subunit A
Ciclev10033044m.g	-1.639914	no_ann
Ciclev10033481m.g	-1.639506	Protein phosphatase 2A regulatory B subunit family protein
Ciclev10032203m.g	-1.598694	NAC domain containing protein 42
Ciclev10002852m.g	-1.581125	no_ann
Ciclev10030694m.g	-1.579975	Pre-mRNA-splicing factor 3
Ciclev10026217m.g	-1.577727	Chaperone DnaJ-domain superfamily protein
Ciclev10016980m.g	-1.575477	no_ann
Ciclev10020800m.g	-1.519573	hydroxyproline-rich glycoprotein family protein
Ciclev10031202m.g	-1.514092	cyclic nucleotide gated channel 1
Ciclev10014200m.g	-1.506895	auxin response factor 8
Ciclev10016516m.g	-1.506755	SNF7 family protein
Ciclev10021832m.g	-1.501381	no_ann
Ciclev10030632m.g	-1.488469	Leucine-rich repeat receptor-like protein kinase family protein
Ciclev10013643m.g	-1.485334	RING/U-box superfamily protein
Ciclev10016894m.g	-1.484521	CAX interacting protein 1
Ciclev10033271m.g	-1.475072	no_ann
Ciclev10033626m.g	-1.460744	Cupredoxin superfamily protein
Ciclev10014605m.g	-1.456247	DNA GYRASE A
Ciclev10032582m.g	-1.449737	Protein of unknown function (DUF1997)
Ciclev10031766m.g	-1.449314	phosphate transporter traffic facilitator1
Ciclev10032240m.g	-1.443933	vitamin E pathway gene 5
Ciclev10004767m.g	-1.441325	Transducin/WD40 repeat-like superfamily protein
Ciclev10018572m.g	-1.436706	disease resistance protein (TIR-NBS-LRR class), putative
Ciclev10014356m.g	-1.435574	Leucine-rich repeat protein kinase family protein
Ciclev10033407m.g	-1.374079	Phosphorylase superfamily protein
Ciclev10015919m.g	-1.370387	Mo25 family protein
Ciclev10032332m.g	-1.360649	no_ann
Ciclev10021520m.g	-1.355703	RNA-binding (RRM/RBD/RNP motifs) family protein
Ciclev10027438m.g	-1.350609	Subtilase family protein
Ciclev10002101m.g	-1.344087	ATP-dependent protease La (LON) domain protein
Ciclev10018118m.g	-1.344082	no_ann
Ciclev10008125m.g	-1.341221	cinnamate-4-hydroxylase
Ciclev10019576m.g	-1.326708	Protein kinase superfamily protein

Ciclev10012685m.g	-1.316716	Phosphatidic acid phosphatase (PAP2) family protein
Ciclev10001047m.g	-1.315723	phospholipid/glycerol acyltransferase family protein
Ciclev10024209m.g	-1.315633	NmrA-like negative transcriptional regulator family protein
Ciclev10024563m.g	-1.292381	S-adenosyl-L-methionine-dependent methyltransferases superfamily protein
Ciclev10033561m.g	-1.290674	SKU5 similar 3
Ciclev10022415m.g	-1.277483	RNA-binding (RRM/RBD/RNP motifs) family protein
Ciclev10003806m.g	-1.264889	SH3 domain-containing protein
Ciclev10033807m.g	-1.263222	Shugoshin C terminus
Ciclev10011875m.g	-1.262731	Ribosomal protein L4/L1 family
Ciclev10020911m.g	-1.261804	trigalactosyldiacylglycerol 1
Ciclev10030689m.g	-1.256038	RING/U-box superfamily protein
Ciclev10007292m.g	-1.245475	Transcriptional factor B3 family protein / auxin-responsive factor AUX/IAA-related
Ciclev10016386m.g	-1.232672	no_ann
Ciclev10026609m.g	-1.214269	chloroplast-localized ISCA-like protein
Ciclev10030782m.g	-1.207382	ABC-2 type transporter family protein
Ciclev10033554m.g	-1.195896	cation/H+ exchanger 2
Ciclev10011191m.g	-1.179486	RHO guanyl-nucleotide exchange factor 7
Ciclev10001111m.g	-1.165314	HXXXD-type acyl-transferase family protein
Ciclev10010277m.g	-1.149876	Outward rectifying potassium channel protein
Ciclev10021850m.g	-1.139984	Protein of unknown function (DUF3755)
Ciclev10000727m.g	-1.137129	P-loop containing nucleoside triphosphate hydrolases superfamily protein
Ciclev10000783m.g	-1.130538	SKU5 similar 5
Ciclev10013362m.g	-1.111221	TTF-type zinc finger protein with HAT dimerisation domain
Ciclev10007954m.g	-1.109791	acetyl Co-enzyme a carboxylase biotin carboxylase subunit
Ciclev10030523m.g	-1.107232	P-loop containing nucleoside triphosphate hydrolases superfamily protein
Ciclev10001333m.g	-1.094804	basic region/leucine zipper transcription factor 16
Ciclev10011222m.g	-1.090246	Leucine-rich repeat receptor-like protein kinase family protein
Ciclev10024316m.g	-1.088006	RING/U-box superfamily protein
Ciclev10033523m.g	-1.078879	Protein kinase family protein with leucine-rich repeat domain
Ciclev10021666m.g	-1.076377	RNA-binding ASCH domain protein
Ciclev10031452m.g	-1.07529	transferases, transferring acyl groups
Ciclev10025951m.g	-1.070489	Tetratricopeptide repeat (TPR)-like superfamily protein
Ciclev10017724m.g	-1.062373	Ubiquitin-like superfamily protein
Ciclev10000836m.g	-1.059386	TCP-1/cpn60 chaperonin family protein
Ciclev10033555m.g	-1.05066	Serine carboxypeptidase S28 family protein
Ciclev10033583m.g	-1.050125	Leucine-rich repeat receptor-like protein kinase family protein
Ciclev10017107m.g	-1.04676	no_ann
Ciclev10031337m.g	-1.043031	Pectin lyase-like superfamily protein
Ciclev10010625m.g	-1.028088	high-affinity K+ transporter 1
Ciclev10005275m.g	-1.024998	O-methyltransferase family protein
Ciclev10000411m.g	-1.010508	Subtilisin-like serine endopeptidase family protein
Ciclev10024314m.g	-1.010459	Leucine-rich repeat transmembrane protein kinase

Table S2. Gene Ontology (GO) categories with statistical significance overrepresented in genes potentially involved in mandarin domestication based on negative Tajima's D values

GO Category	adj P-value
arabinan catabolic process(GO:0031222)	4.31E-02
regulation of multicellular organism growth(GO:0040014)	3.47E-02

borate transport(GO:0046713)	2.14E-02
cadmium ion transmembrane transport(GO:0070574)	2.92E-02
plant-type primary cell wall biogenesis(GO:0009833)	1.52E-02
protein monoubiquitination(GO:0006513)	4.07E-02
cellular iron ion homeostasis(GO:0006879)	3.62E-03
monocarboxylic acid metabolic process(GO:0032787)	4.17E-02
maintenance of shoot apical meristem identity(GO:0010492)	2.59E-02
water homeostasis(GO:0030104)	4.33E-02
developmental process involved in reproduction(GO:0003006)	3.66E-02
detection of bacterium(GO:0016045)	4.31E-02
methane metabolic process(GO:0015947)	1.78E-02
triglyceride biosynthetic process(GO:0019432)	3.43E-02
programmed cell death(GO:0012501)	1.87E-02
lysine biosynthetic process via diaminopimelate(GO:0009089)	3.70E-02
Mo-molybdopterin cofactor biosynthetic process(GO:0006777)	4.18E-02
histone H3-K4 methylation(GO:0051568)	4.21E-02
tricarboxylic acid cycle(GO:0006099)	7.21E-03
regulation of macromolecule metabolic process(GO:0060255)	2.83E-02
lignin catabolic process(GO:0046274)	9.70E-03
regulation of pH(GO:0006885)	2.90E-04
actin filament-based movement(GO:0030048)	1.40E-02
lateral root formation(GO:0010311)	2.95E-03
mitochondrial transport(GO:0006839)	3.67E-02
chromatin silencing(GO:0006342)	1.07E-03
chromatin organization(GO:0006325)	4.07E-02
floral organ abscission(GO:0010227)	3.91E-02
pectin catabolic process(GO:0045490)	2.02E-02
glycine metabolic process(GO:0006544)	3.58E-03
threonine metabolic process(GO:0006566)	6.28E-03
L-serine metabolic process(GO:0006563)	8.29E-03
gene silencing by RNA(GO:0031047)	3.45E-02
calcium ion transport(GO:0006816)	4.18E-02
chloroplast organization(GO:0009658)	2.10E-02
hydrogen ion transmembrane transport(GO:1902600)	8.88E-03
acyl-carrier-protein biosynthetic process(GO:0042967)	1.70E-03

Table S3. Gene Ontology (GO) categories with statistical significance overrepresented in genes involved in mandarin domestication based on high PBS values

GO category	adj P-value
lipid particle organization(GO:0034389)	2.61E-02
glucose-6-phosphate transport(GO:0015760)	2.71E-02
copper ion export(GO:0060003)	3.12E-02
photosynthetic acclimation(GO:0009643)	1.68E-02
phosphoenolpyruvate transport(GO:0015714)	4.09E-02
triose phosphate transmembrane transport(GO:0035436)	4.02E-02
gamete generation(GO:0007276)	2.39E-02
methylation(GO:0032259)	1.96E-03
response to arsenic-containing substance(GO:0046685)	1.34E-02
negative regulation of flower development(GO:0009910)	3.15E-02
vernalization response(GO:0010048)	2.48E-02
response to gibberellin(GO:0009739)	3.62E-02
production of miRNAs involved in gene silencing by miRNA(GO:0035196)	4.63E-02
defense response(GO:0006952)	2.94E-02
regulation of transcription, DNA-templated(GO:0006355)	3.13E-02
